# Production of hyperimmune anti-SARS-CoV-2 intravenous immunoglobulin from pooled COVID-19 convalescent plasma

**DOI:** 10.2217/imt-2020-0263

**Published:** 2021-02-09

**Authors:** Shaukat Ali, Syed M Uddin, Ayesha Ali, Fatima Anjum, Rashid Ali, Elisha Shalim, Mujtaba Khan, Iqra Ahmed, Sheikh M Muhaymin, Uzma Bukhari, Shobha Luxmi, Abdul S Khan, Saeed Quraishy

**Affiliations:** ^1^Dow College of Biotechnology, Dow University of Health Sciences, Karachi, Pakistan; ^2^Dow Research Institute of Biotechnology & Biomedical Sciences, Dow University of Health Sciences, Karachi, Pakistan; ^3^Dow International Medical College, Dow University of Health Sciences, Karachi, Pakistan; ^4^Dow University Hospital, Dow University of Health Sciences, Karachi, Pakistan; ^5^National Control Laboratory for Biologicals, Islamabad, Pakistan; ^6^Dow University of Health Sciences, Karachi, Pakistan

**Keywords:** convalescent plasma, SARS-CoV-2, intravenous immunoglobulins, anti-COVID-19 IVIG, passive immunization, pooled plasma, caprylic acid, ultrafiltration, diafiltration, immunotherapy

## Abstract

**Background:** This study assesses the feasibility of producing hyperimmune anti-COVID-19 intravenously administrable immunoglobulin (C-IVIG) from pooled convalescent plasma (PCP) to provide a safe and effective passive immunization treatment option for COVID-19. **Materials & methods:** PCP was fractionated by modified caprylic acid precipitation followed by ultrafiltration/diafiltration to produce hyperimmune C-IVIG. **Results:** In C-IVIG, the mean SARS-CoV-2 antibody level was found to be threefold (104 ± 30 cut-off index) that of the PCP (36 ± 8.5 cut-off index) and mean protein concentration was found to be 46 ± 3.7 g/l, comprised of 89.5% immunoglobulins. **Conclusion:** The current method of producing C-IVIG is feasible as it uses locally available PCP and simpler technology and yields a high titer of SARS-CoV-2 antibody. The safety and efficacy of C-IVIG will be evaluated in a registered clinical trial (NCT 04521309).

In late 2019, an outbreak of severe acute respiratory syndrome coronavirus-2 (SARS-CoV-2) occurred in China with an enduring risk of pandemic [1]. SARS-CoV-2 is an enveloped positive sense RNA virus that is widely distributed in humans, mammals and birds and causes a respiratory, liver, neurological and intestinal illness referred to as COVID-19 [2]. In general it is an acute resolved infection, but in some cases it can be fatal and results in death due to massive alveolar damage that leads to respiratory failure [3].

As of 3 December 3 2020, more than 63.7 million cases were laboratory confirmed and over 1.4 million deaths had been reported globally due to COVID-19 [4]. The disease poses a great threat to health and there has already been considerable socioeconomic damage to countries globally [5]. Social distancing and lockdown measures have been adopted worldwide to control infection spread, minimize the number of active cases and subsequently reduce the death rate in most affected areas.

Until now, no approved therapeutic option specifically against COVID-19 is available; however, supportive therapy that is given in healthcare settings includes oxygenation, fluid management and ventilation [6]. Efforts are being carried out by several biotechnological and pharmaceutical companies for the delivery of safe and effective vaccine that is accessible globally [7].

In the absence of a formal effective treatment and widespread availability of immunization, scientific literature advocated the use of passive immunization strategies for treating COVID-19 based on their effectiveness in SARS, Middle East respiratory syndrome and influenza outbreaks [8]. In March 2020, the US FDA approved the use of convalescent plasma therapy for patients infected with SARS-CoV-2 on a conditional basis [9], which led to the registration of multiple clinical trials globally, and the first evidence that anti-SARS-CoV-2 antibodies present in plasma could effectively treat COVID-19 patients came from the work of Cheng *et al.* [10]. However, reports regarding the safety and efficacy of convalescent plasma in COVID-19 infection are still inconclusive due to limited sample size, study design, early termination of the trial and/or unavailability of results [11]. High doses of nonspecific intravenously administrable immunoglobulin (IVIG) are reported to improve recovery in severe cases (when given at an early stage of the disease) by suppressing inflammatory markers through immune modulation [12,13]. Polyclonal and monoclonal immunoglobulin preparations have been used for prophylaxis and treatment of viral infections such as respiratory syncytial virus infection and human CMV infection [14]. Polyclonal anti-COVID-19 IVIG (C-IVIG) is therefore expected to be an effective approach for treating COVID-19 patients by neutralizing SARS-CoV-2, limiting its dissemination inside the patient and thus limiting the course of disease [15].

Caprylic acid-based plasma fractionation is a relatively simple and practical method that can be used even in technologically less advanced countries in this time of pandemic for widespread availability of hyperimmune C-IVIG. Caprylic acid-based protein purification has been successfully applied for commercial production of equine plasma-derived antivenoms [16]. El-Ekiaby *et al*. reported fractionation of human plasma minipools using caprylic acid precipitation to produce IgG preparations [17]. Due to fewer processing steps, robust viral inactivation, expected high purity and product yield, caprylic acid precipitation is expected to be an ideal method for production of safe and effective C-IVIG.

This study aims to demonstrate the feasibility of the modified caprylic acid method for C-IVIG production from pooled convalescent plasma (PCP) of COVID-19 recovered individuals.

## Materials & methods

### Donor selection & plasma collection

Convalescent plasma was collected from consenting COVID-19 recovered individuals meeting the inclusion criteria: prior diagnosis of COVID-19 documented by a laboratory test, complete resolution of symptoms at least 14 days prior to donation, female donors negative for HLA antibodies, or male donors. All donors were screened for transfusion-transmitted infections (HIV, HBV, HCV, malaria and syphilis) and total SARS-COV-2 antibody titer. None of the donors had previously donated convalescent plasma and they did not donate again for the study. The blood bank is licensed by the regional blood transfusion authority, and the procedure was approved by Institutional Review Board (IRB-1685/DUHS/Approval/2020/).

Convalescent plasma (600–800 ml) was collected by plasmapheresis using a therapeutic apheresis machine (COBE Spectra, Terumo Medical, CO, USA). Collected plasma was aseptically transferred into multiple units for storage at -30°C.

Pre-pandemic fresh frozen plasma (collected before November 2019) was also processed to produce control IVIG.

### Preparation of PCP

A PCP batch of 4 ± 0.1 or 8 ± 0.1 l was prepared for subsequent processing by thawing (at 30°C) and pooling frozen convalescent plasma bags from 16–20 individual donors into depyrogenated glass containers under a laminar flow hood. The anti-SARS-CoV-2 antibody titer and concentration of IgG, IgA, IgM and albumin in the pooled plasma was measured before processing.

### Caprylic acid precipitation

Precipitation of plasma proteins other than immunoglobulin was carried out in a vertical laminar flow unit by using the caprylic acid precipitation method with modification [18]. The pH was adjusted to 6.0 with 3N HCl and then 5% v/v caprylic acid was added slowly to the PCP with constant vigorous stirring. The vigorous stirring was continued for 1 h at 25°C and pH ∼5.0, after which precipitated proteins were removed by centrifugation at 11,000 × g for 30 min at 4°C. The supernatant was filtered before proceeding to ultrafiltration/diafiltration for further purification.

### Ultrafiltration/diafiltration

After precipitation, ultrafiltration/diafiltration of the supernatant was performed using a 100-kDa ultrafiltration cassette (Biomax, Merck, NJ, USA) to concentrate immunoglobulin and remove impurities like caprylic acid. The supernatant was aseptically transferred into the feed reservoir of the ultrafiltration equipment (ÄKTA Flux 6, GE, Uppsala, Sweden) in a laminar flow unit and ultrafiltration/diafiltration was performed at 15 psi pressure drop (ΔP) and transmembrane pressure was manually adjusted at 15 psi. Diafiltration with pyrogen-free formulation buffer (50 mg/ml dextrose and 77mM NaCl) was performed using six diavolumes and the volume of the retentate was reduced to achieve 5× concentration. The concentrated retentate was collected into a depyrogenated glass container after terminal filtration with 0.2 μm sterile filters (Sartobran, Sartorius, Goettingen, Germany).

### Protein quantification & purity profile

Total protein was quantified by automated laboratory system Atellica^®^ chemistry analyzer (Siemens, NY, USA) using the biuret method. The purity profile of the finished product was determined by SDS-PAGE. According to Laemmli (16), stacking (4%) and resolving (10%) gels were prepared [19]. The samples were run under reducing and nonreducing conditions, and protein bands were visualized after staining with Coomassie brilliant blue. A reference range marker of 10 to 250 kDa (PageRuler™ Plus Prestained Protein Ladder 10 to 250 kDa, Thermo Scientific, MA, USA) was used.

### IgG, IgM, IgA & albumin measurement

The total IgG, IgM, IgA in pooled plasma, control IVIG and C-IVIG were measured by immunoturbidimetry using the Atellica^®^ chemistry analyzer. Albumin was measured by the bromocresol purple dye-binding method using ARCHITECT Systems^®^ (Abbott, IL, USA). Samples that exceeded the detection limit were diluted accordingly.

### SARS-CoV-2 antibody titer measurement

Electrochemiluminescence immunoassay was performed for determination of anti-SARS-CoV-2 antibody titer using Elecsys^®^ Anti-SARS-CoV-2 kit (Roche Diagnostics, Basel, Switzerland). The assay is based on the double antigen principle, whereby biotinylated SARS-CoV-2-specific recombinant nucleocapsid antigen and ruthenium-labelled SARS-CoV-2-specific recombinant antigen form a sandwich complex with SARS-CoV-2-specific antibodies. Blood of plasma donor was collected in an acid citrate dextrose vial and centrifuged to collect plasma. The tube containing 1–2 ml plasma was placed in automated electrochemiluminescence analyzer (Cobas e601) for reagent mixing, reaction processing and signal recording. For C-IVIG anti-SARS-CoV-2, antibodies were measured using a 1:4 dilution to avoid any assay limitation. Results were reported as cut-off index (COI), which is based on the ratio of assay signal to cut-off signal (cut-off = 1).

### Bioburden analysis

The final product was tested for any potential bioburden. Product sterility was determined in accordance with US pharmacopeia. Tryptic soy broth and fluid thioglycollate medium (Merck, MA, USA) were used and 10% of the sample was inoculated in each of the growth medium (USP-71) [20].

### Rabbit pyrogen testing

Healthy rabbits weighing 0.8–1 kg were used. An infusion of 0.03 g/ml was prepared and a dose of 0.15–0.2 g/kg was administered into a marginal vein of the ear of each rabbit. Animals were observed for 3 h and temperatures were recorded at intervals of 30 min (USP-151) [21].

### *In vivo* toxicity assessment

Male Wistar rats of 220–260 g were used after a period of acclimatization. The animals used in this study were maintained in accordance with the ethical guidelines of Dow University of Health Sciences, Karachi, Pakistan.

**Figure 1.**
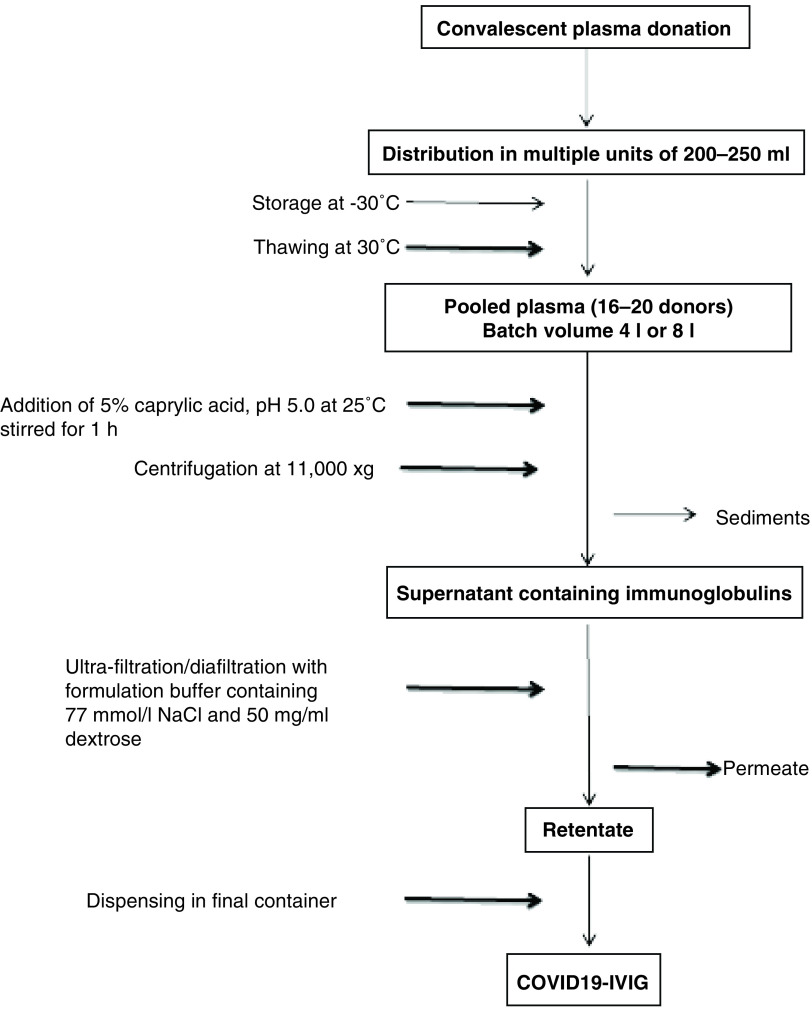
Process flow diagram for the production of high-titer anti-SARS-CoV-2 intravenously administrable immunoglobulin. IVIG: Intravenously administrable immunoglobulin.

Animals were divided into three groups of three rats each. IVIG was administered intravenously and dosage was adjusted according to body weight. The first and second groups were considered as low and high dose, respectively (low dosage: 0.3 g/kg; high dosage: 0.5 g/kg) and the control group was administered with saline. All groups were observed daily for behavioral and physical changes for 7 days and then were sacrificed. Blood, liver and kidney samples were collected for hematological and histopathological analysis. Figure 1 shows the process flow diagram for the production of high-titer C-IVIG.

## Results

### Immunoglobulin concentration

**Figure 2.**
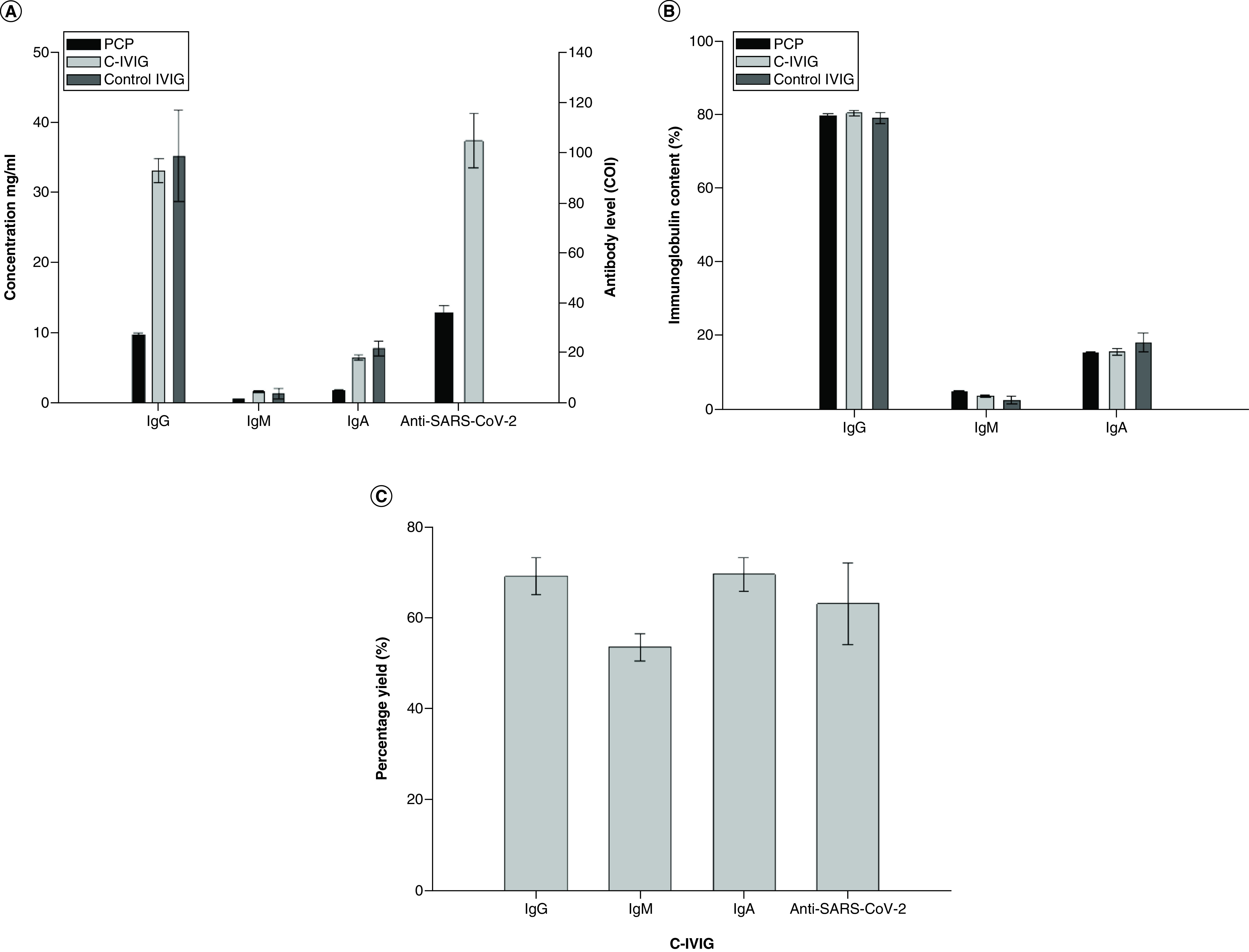
Immunoglobulin content and anti-SARS-CoV-2 antibody level. **(A)** Comparison of mean IgG, IgM, IgA and anti-SARS-CoV-2 antibody levels in pooled convalescent plasma with that in C-IVIG and control IVIG produced by the caprylic acid precipitation method. Control IVIG (pre-pandemic) was found to be nonreactive for SARS-CoV-2 antibodies. The anti-SARS-CoV-2 antibody level is measured as cut-off index (sample absorbance/cut-off; cut-off = 1). **(B)** Comparison of IgG, IgM and IgA content (as percentage of total immunoglobulin) in pooled plasma, C-IVIG and control IVIG. **(C)** Mean percentage yield of IgG, IgA, IgM and anti-SARS-CoV-2 antibodies from convalescent plasma fractionation. All values are represented as the mean of eight consecutive batches of C-IVIG production. C-IVIG: Anti-COVID-19 intravenously administrable immunoglobulin; IVIG: Intravenously administrable immunoglobulin.

The concentrations of IgG, IgM and IgA were measured in C-IVIG, control IVIG and PCP to compare immunoglobulin composition. The mean concentrations of IgG, IgM and IgA in C-IVIG were found to be 33 ± 4.8, 1.6 ± 0.4 and 6.5 ± 1.0 g/l, respectively (Figure 2A), while the mean total protein concentration of all batches was found to be 45.9 ± 3.68 g/l, comprising 89.5% immunoglobulin (Table 1). The mean concentrations of IgG, IgM and IgA in PCP were found to be 9.60 ± 0.8, 0.62 ± 0.1 and 1.86 ± 0.1 g/l, respectively (Figure 2A). The percentage composition of immunoglobulins was found to be similar in PCP, C-IVIG and control IVIG: IgG ∼80%, IgM ∼5% and IgA ∼15% (Figure 2B). The method applied for PCP fractionation resulted in process yields of 69% (IgG), 53% (IgM) and 70% (IgA) (Figure 2C).

**Table 1.** Characteristics of the anti-COVID-19 intravenously administrable immunoglobulin 5% solution.

Parameters	Batch 1	Batch 2	Batch 3	Batch 4	Batch 5	Batch 6	Batch 7	Batch 8	Mean ± standard deviation
Formulation	Liquid	Liquid	Liquid	Liquid	Liquid	Liquid	Liquid	Liquid	–
Appearance	Clear, blue opalescent	Clear, blue opalescent	Clear, blue opalescent	Clear, blue opalescent	Clear, blue opalescent	Clear, blue opalescent	Clear, blue opalescent	Clear, blue opalescent	–
pH	6.75	6.62		6.57	6.49	6.78	7.04	6.62	6.7 ± 0.17
Osmolality (mOsmol/kg)	481.5	427.5	447.5	427.5	413.5	411.5	408	409	428.2 ± 25.3
Total proteins (g/l)	42	45	50	44	43	43	48	52	45.9 ± 3.7
IgG (%)	79.8	81.8	77.7	78.6	79.7	79	80.8	83.8	80.1 ± 1.9
IgA (%)	15.5	15.2	18.4	17.6	17.0	17.3	14.2	11.5	15.8 ± 2.2
IgM (%)	4.7	3.0	3.9	3.8	3.3	3.6	5.0	4.7	4.0 ± 0.7
Albumin (g/dl)	<0.4	<0.4	<0.4	<0.4	<0.4	<0.4	<0.4	<0.4	^†^
Anti-SARS-CoV-2 (COI)	111.1	131.3	140	122.1	46.5	106.9	77.6	99.6	104.4 ± 30.4
Pyrogen test	passed	passed	passed	passed	passed	passed	passed	passed	–
Sterility	passed	passed	passed	passed	passed	passed	passed	passed	–

† Below detection limit.

COI: Cut-off index.

### Anti-SARS-CoV-2 antibody level

The mean level of anti-SARS-CoV-2 antibodies in C-IVIG of eight batches was found to be 104.3 ± 30.3; this was threefold higher than the mean anti-SARS-CoV-2 antibody level in the PCP used for production of batches. The control IVIG produced from prepandemic plasma was found to be nonreactive to SARS-CoV2 antibody. The mean process yield for SARS-CoV2 antibody was found to be 59 % (Figure 2C).

### Product safety assessment

**Figure 3.**
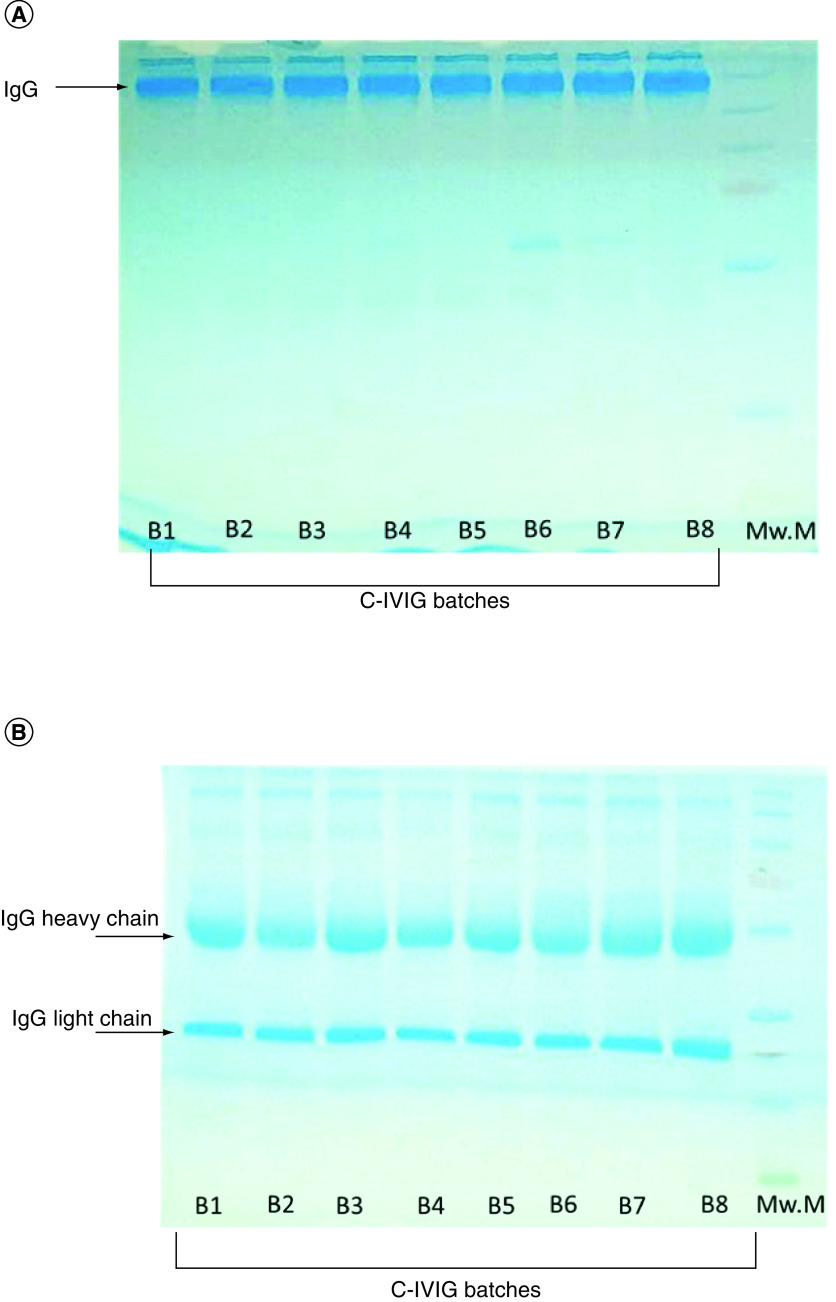
SDS-PAGE. Patterns of eight consecutive batches of IVIG in **(A)** non-reducing and **(B)** reducing conditions. B1–B8: Batch numbers; IVIG: Intravenously administrable immunoglobulin; Mw.M: Molecular weight markers (10–250 KDa).

Each batch of final IVIG product was found to be sterile with no sign of turbidity observed in any liquid culture tube after 14 days of incubation at respective temperatures. SDS-PAGE analysis with 10% separating gel under nonreducing conditions showed a predominant band around 150–160 kDa, confirming the presence of IgG with monomeric IgA. Similarly, under nonreducing conditions two bands of around 50 and 25 kDa were observed, representing the heavy and light chains of IgG (Figure 3). Rabbit pyrogen tests for all batches were cleared. No fever or significant rise in temperature was observed in any of the rabbits, and there were no anaphylactic reactions. Rabbits were observed for a further 1 week, and no mortality was observed.

### Toxicity test

Toxicity testing did not show any mortality or behavioral changes in any of the rat groups. No significant difference was observed in their body weights (Supplementary Table 2). Food and water consumption was not remarkably different over the 7-day observation period post-infusion, after which rats were sacrificed.

### Histopathological examination

**Figure 4.**
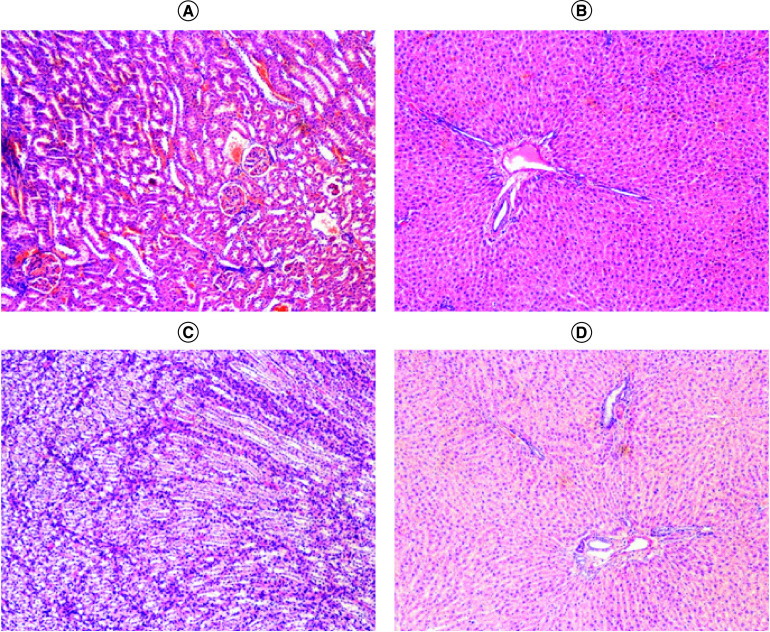
Histological examination of liver and kidney of rats. **(A)** Cross-section of kidney treated with 0.5 mg/kg dose of intravenously administrable immunoglobulin. **(B)** Cross-section of liver treated with 0.5 mg/kg dose. **(C)** Cross-section of kidney of control group. **(D)** Cross-section of liver of control group.

Histopathological examination (Figure 4) of liver from animals treated with two different doses (0.3 and 0.5 g/kg) of IVIG showed no distortion of cells; that is, the findings were similar to those in the control group. Moreover, there were no signs of injury, fatty acid accumulation or hemorrhagic regions around the central vein or sinusoids of the liver. Arrangement of hepatocytes around the cords was visible. Cross-sections of liver revealed that there was no infiltration of lymphocytes, neutrophils, or macrophages and that none of the blood cells were lysed. In the kidneys, mild vascular congestion was observed in both test and control groups. However, no evidence of necrosis, inflammation, atypia or any significant pathological change was seen.

### Hematological assays

Hematological assays showed no significant changes in most of the parameters, apart from an eosinophil count higher than the standard range; however, increased levels of Mean corpuscular volume (MCV) and lymphocytes were observed in all the studied groups, including control group (Supplementary Table 3).

### Biochemical parameters

Liver function tests of all the tested groups showed slight increases in direct bilirubin and alkaline phosphatase levels when compared with the standard range; these increased levels were also observed in the control group that was only infused with normal saline (Supplementary Table 4).

## Discussion

Although limited data are available regarding mechanisms of immune protection against SARS-CoV-2, it is believed that anti-SARS-CoV-2 neutralizing antibodies play a crucial role. Passive immunization in general, and hyperimmune IVIG in particular, is used against a few infectious diseases [22], especially in the case of life-threatening situations and novel viral outbreaks such as SARS-CoV-2, where there is an absence of licensed medication or a specific treatment modality [12,23]. Currently, convalescent plasma and nonspecific IVIG are under trial [24], and a safe and effective treatment through hyperimmune IVIG is of great interest [25].

In this study, C-IVIG was prepared using convalescent plasma mostly obtained from recovered individuals who had mild or moderate symptoms, while severely ill patients were avoided due to donor health concerns. As reported by Weidner *et al*. [26], donors were found to be reactive for antibodies with highly variable antibody titers due to being at different stages of recovery. Pooling convalescent plasma from 16–20 donors not only provides consistent raw material despite variability in individual plasma, but would also be expected to enhance polyvalency of neutralizing antibodies, which is expected to increase effectiveness of the C-IVIG. However, the polyvalency of C-IVIG in particular has not been proven in this study. The selection of local plasma donors may also provide antibodies against a variety of indigenous SARS-CoV-2 strains, instead of using monoclonal antibodies that provide protection against one specific strain of virus.

Unlike commercially predominant methods for IVIG production, such as cold ethanol fractionation [27], the C-IVIG product development strategy detailed in this study involved limited processing steps and simpler techniques. This ensures practicality and feasibility of the manufacturing process to address the urgent global need for widespread availability during the COVID-19 pandemic. A combination of caprylic acid precipitation and tangential flow filtration led to the preparation of high-titer C-IVIG. Consistency and robustness of the product in eight consecutive batches was indicated after analysis of IgG, IgM and IgA concentrations. Owing to process losses at different stages of production, an overall process yield of 68% was achieved, comparable to that of similar processes reported by Morais *et al.* and El-Ekiaby *et al*. [17,18]. The percentage composition of immunoglobulin in C-IVIG and PCP (80% IgG, 15% IgA and 5% IgM) was found to be similar to that in serum of normal healthy individuals [28–30], suggesting efficiency of the production process and physiological suitability of the product. One of the limitations in this work was the failure to minimize IgA concentration in C-IVIG [31] for compatibility with IgA deficient patients, through inclusion of further purification steps. However, given that IgA can serve as a SARS-CoV-2-neutralizing antibody [32], the inclusion of further steps would decrease the yield of the process where availability of convalescent plasma was already a limitation.

The use of 5% caprylic acid for precipitation in this method also ensures the inactivation of enveloped viruses, as it is known to disrupt the lipid envelope and inactivate enveloped viruses completely in immunoglobulin solutions [17]. Ultrafiltration/diafiltration is used to remove residual caprylic acid from solution after caprylic acid precipitation, followed by the addition of formulation buffer containing stabilizers and purification and concentration of the solution to the desired 5% preparation [33–35].

Due to unavailability of SARS-CoV-2 neutralization assays, the SARS-CoV-2 antibody titer was determined using the Elecsys^®^ kit. The kit is reported to have sensitivity of 95% and it showed significant correlation with neutralization test (NT) titers [26]; hence it was used as an alternative to NT for donor screening and titer measurement in PCP and C-IVIG. However, using Elecsys is a limitation in our study and is not recommended when NT or other strongly correlated tests are available. The mean titer of 104 COI is considered high compared with the titer in convalescent plasma collected for this study, which had a mean of 36 COI and reached a maximum of 60 COI for an individual donor. Control IVIG prepared from pre-pandemic plasma was found to be nonreactive for SARS-CoV-2 antibodies, consistent with data reported by Yang *et al.* [36], so it can be deduced that commercially available nonspecific IVIG made in the prepandemic era will not be able to neutralize SARS-CoV-2.

The formulation buffer used in C-IVIG preparation contains dextrose and sodium chloride (50 g/l dextrose and 77mM NaCl). The dextrose serves as a stabilizing agent for immunoglobulin at pH >4.5, as in the case of C-IVIG where pH was maintained around physiological pH (∼7). The sodium concentration in the buffer was comparable to that of commercial IVIG preparations, to form a hypotonic solution (0.45% saline) [31]. Hence the osmolality of C-IVIG was also within the range of commercially available IVIG products [37]. Safety of the formulation was further tested on rats to observe any toxic, pathological or adverse effects *in vivo*. Rabbit pyrogen test was used instead of the Limulus amebocyte lysate assay to check for pyrogenicity of C-IVIG, due to interfering factors in blood-derived products that limit bacterial endotoxin measurement through the Limulus amebocyte lysate assay [38]. Although the reuse of animals for rabbit pyrogen tests is common, it is not recommended for immunogenic preparations; hence rabbits used once were not used again. Product stability tests are being conducted, however sufficient data are not currently available.

## Conclusion

With multiple treatment modalities against COVID-19 still under trial, this study aimed to develop a method based on passive immunization through IVIG produced from COVID-19 convalescent plasma collected locally. The C-IVIG production method applied in this study is feasible, practical and could be implemented at local level using indigenously collected convalescent plasma against local SARS-CoV-2 strains. The process resulted in a product that was safe, effective and physically comparable to commercially available IVIG. The 5% C-IVIG solution comprised 90% immunoglobulin (IgG, IgM and IgA) with a total anti-SARS-CoV-2 antibody titer three-times higher than in PCP, which makes this treatment potentially more effective than the other passive immunization methods available. The C-IVIG will be used in a registered clinical trial (NCT 04521309) for further evaluation of its safety and efficacy.

**Summary points**

- SARS-CoV-2 infection has emerged as a global health crisis with no particular licensed treatment available at the time of writing.
- The effectiveness of passive immunization using convalescent plasma and nonspecific intravenously administrable immunoglobulin is yet to be established for COVID-19.
- Experts believe hyperimmune intravenous immunoglobulin preparation can be a safe and effective therapeutic option against COVID-19 until an approved vaccine or medication becomes available.
- In this study convalescent plasma from COVID-19 recovered individuals was collected through plasmapheresis, then pooled and fractionated by a modified caprylic acid precipitation method to produce anti-COVID-19 intravenous immunoglobulin (C-IVIG).
- The C-IVIG preparation is a liquid formulation that can be administered intravenously into COVID-19 infected individuals in early stages to neutralize SARS-CoV-2.
- C-IVIG is prepared by caprylic acid precipitation of pooled COVID-19 convalescent plasma, followed by ultrafiltration/diafiltration to concentrate immunoglobulins.
- Analytical assessment of C-IVIG confirmed its safety.
- The product has been registered for clinical trials to evaluate its safety and efficacy in patients with SARS-CoV-2 infection.
