# Supplementary material for: Production of hyperimmune anti-SARS-CoV-2 intravenous immunoglobulin from pooled COVID-19 convalescent plasma
Source: Immunotherapy. 2021 Feb 9:10.2217/imt-2020-0263. doi: 10.2217/imt-2020-0263 (PMC7871744; doi:10.2217/imt-2020-0263)
Supplement: Supplementary file 1 [file supplementary_data_tables.docx]

Table 2: Mean body weight of rats before and after IVIG infusion

| Treatment groups | 0 day | After 7 days |
| --- | --- | --- |
| Control | 244.7±16.5 | 253.33±5.033 |
| Low dose | 246.7±10.7 | 260.83±111.10 |
| High dose | 237.3±17.2 | 241.5±17.77 |

| Tests | Control | High dose | Low dose | Range |
| --- | --- | --- | --- | --- |
| Total Bilirubin (mg/dL) | 0.13±0.02 | 0.13±0 | 0.128±0.007 | 0.05-0.15 |
| Direct Bilirubin(mg/dL) | 0.1±0 | 0.1±0 | 0.1±0 | 0.03-0.05 |
| Indirect Bilirubin (mg/dL) | 0.03±0.02 | 0.03±0 | 0.02±0.01 | 0.01-0.12 |
| SGPT(ALT) (U/L) | 55.33±9.29 | 51.83±5.41 | 50.665±6.59 | 7-56 |
| Alkaline Phosphatase (U/L) | 274±52.37 | 288.66±42.42 | 265.65±15.06 | 62-230 |
| Gamma GT(U/L) | 4±0 | 4±0 | 4±0 | 9-48 |
| SGOT(AST) (U/L) | 188±48.12 | 158.37±14.375 | 173.15±12.94 | 5-40 |

Table 3: Hematological parameters of rats infused with different doses of IVIG

Data were expressed as mean±SD

Table 4: Biochemical parameters of rats infused with different doses of IVIG

| **Parameters** | **Control** | **High dose** | **Low dose** | **Range** |
| --- | --- | --- | --- | --- |
| HB (g/dL) | 12.1±3.25 | 13.41±0.07 | 13.34±0.02 | 13.7-17.6 |
| RBC (10^12^/L) | 6.6±1.83 | 7.433±0.14 | 7.29±0.33 | 1.96-8.25 |
| HCT (%) | 41±9.89 | 44.665±0.47 | 44.5±0.70 | 39.6-52.5 |
| MCV (fL) | 64±3.26 | 60.165±0.23 | 61.165±1.64 | 48.9-57.9 |
| MCH (pg) | 18.33±0.47 | 18.165±0.23 | 18.6±0.94 | 17.1-20.4 |
| MCHC (gm/dL) | 29.66±0.47 | 29.995±0.47 | 29.8±0.28 | 32.9-37.5 |
| WBC (10^9^/L) | 6.53±5.20 | 8.765±0.89 | 7.1±3.25 | 1.96-8.25 |
| Platelets (10^9^/L) | 646±531.66 | 798.665±199.8 | 714.6±81.08 | 638-1177 |
| Neutrophils (%) | 31.33±11.01 | 24.495±1.64 | 33.165±13.90 | 6.2-26.7 |
| Lymphocytes (%) | 58.66±9.89 | 63.33±2.82 | 55.33±14.14 | 66.6-90.3 |
| Monocytes (%) | 6.66±2.05 | 5.995±0.47 | 6.83±0.70 | 0.8-3.8 |
| Eosinophils (%) | 3.33±1.15 | 6.165±1.64 | 4.66±0.40 | 0.2-3.5 |
| Basophils (%) | 0 | 0 | 0 | 0-0.8 |

Data were expressed as mean±SD, whereas HB=Hemoglobin, RBC= Red Blood Cells, HCT=Hematocrit, MCV= Mean Corpuscular Volume, MCH=Mean Corpuscular Hemoglobin, MCHC= Mean corpuscular hemoglobin concentration.
